# Highly Emissive Biological Bilirubin Molecules: Shedding New Light on the Phototherapy Scheme

**DOI:** 10.1021/acs.jpcb.1c05308

**Published:** 2021-08-04

**Authors:** Ahmed M. El-Zohry, Valentin Diez-Cabanes, Mariachiara Pastore, Taha Ahmed, Burkhard Zietz

**Affiliations:** † Department of Chemistry, Ångström Laboratories, Box 523, SE-75120 Uppsala, Sweden; ‡ Department of Physics - AlbaNova Universitetscentrum, Stockholm University, SE-10691 Stockholm, Sweden; § Université de Lorraine & CNRS, Laboratoire de Physique et Chimie Théoriques (LPCT), F-54000 Nancy, France

## Abstract

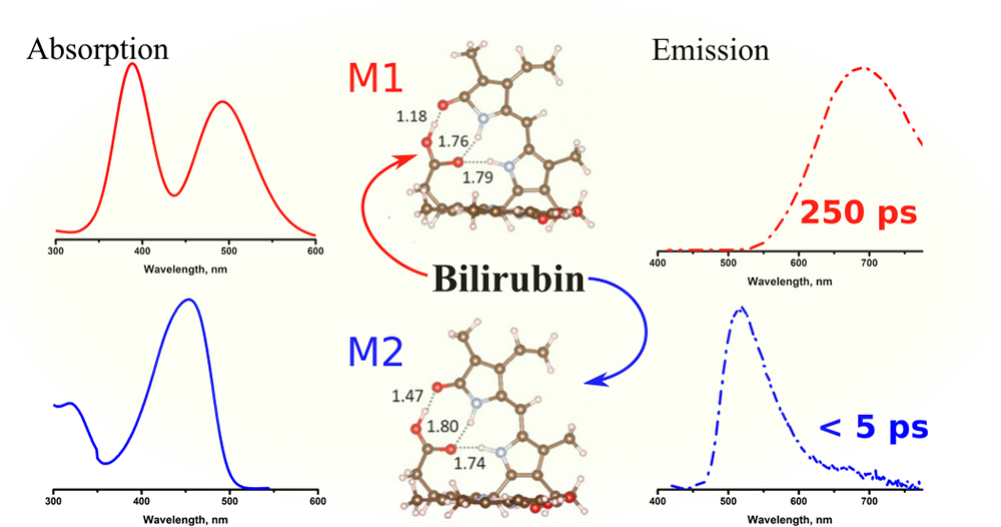

Bilirubin (BR) is the main end-product of the hemoglobin catabolism. For decades, its photophysics has been mainly discussed in terms of ultrafast deactivation of the excited state in solution, where, indeed, BR shows a very low green emission quantum yield (EQY), 0.03%, resulting from an efficient nonradiative isomerization process. Herein, we present, for the first time, unique and exceptional photophysical properties of solid-state BR, which amend by changing the type of crystal, from a closely packed α crystal to an amorphous loosely packed β crystal. BR α crystals show a very bright red emission with an EQY of ca. 24%, whereas β crystals present, in addition, a low green EQY of ca. 0.5%. By combining density functional theory (DFT) calculations and time-resolved emission spectroscopy, we trace back this dual emission to the presence of two types of BR molecules in the crystal: a “stiff” monomer, M1, distorted by particularly strong internal H-bonds and a “floppy” monomer, M2, having a structure close to that of BR in solution. We assign the red strong emission of BR crystals to M1 present in both the α and β crystals, while the low green emission, only present in the amorphous (β) crystal, is interpreted as M2 emission. Efficient energy-transfer processes from M2 to M1 in the closely packed α crystal are invoked to explain the absence of the green component in its emission spectrum. Interestingly, these unique photophysical properties of BR remain in polar solvents such as water. Based on these unprecedented findings, we propose a new model for the phototherapy scheme of BR inside the human body and highlight the usefulness of BR as a strong biological fluorescent probe.

## Introduction

Bilirubin IXα (termed BR hereafter) is among important biological metabolites which undergoes biologically relevant processes through interaction with light, such as rhodopsin (causing vision)^1,2^ and chlorophyll (photosynthesis in plants).^3^ BR is formed mainly from the degradation cycle of hemoglobin, and it is naturally excreted with the help of liver, after binding to human serum albumin (HSA) and other sugar groups.^4−6^ In newborns, however, the liver might not be active for few days after delivery, causing an accumulation of neurotoxic BR, which manifests itself as neonatal jaundice, affecting the brain functionality and leading to death in extreme cases. There is, thus, an urgent necessity to remove the accumulated BR. This is commonly performed by resorting to BR phototherapy, that is, by exposing the patient to irradiation in the visible range.^7^ The current established mechanism for BR excretion is that blue-to-green light converts the insoluble BR isomers, through the isomerization process, into soluble ones that can be excreted.^8^ BR is a highly nonpolar molecule that tends to interact with lipids rather than with physiological water molecules.^9^ Despite several studies about the photodynamics of BR, many processes are not well understood yet because of the complexity of the BR chemical structure.^10−14^

Because of the insolubility of BR in most solvents, the photophysical properties of BR in vitro, or upon interaction with HAS, have been studied mainly in nonpolar or basic media to prepare clear solutions of BR, that is, the soluble form of BR.^15−18^ These photophysical studies indicate that, upon photoexcitation, an efficient ultrafast isomerization takes place, converting the Z,Z-BR isomer into a mixture of Z,E isomers with a reaction quantum yield of 10%, while the rest retains back to the Z,Z-BR isomer.^10,12,19^ In CHCl_3_, BR shows a very weak emission quantum yield (EQY) of 0.03% (τ_obs_ ∼ 0.3 ps, τ_r_ ∼ 1.0 ns), which increases to 0.3% upon binding to HAS that restricts the dominating isomerization process of soluble BR.^12,20^

Despite the importance of the previous studies, we think this is not the best way to obtain biologically relevant insights into the BR photophysics because it is not really connected to the expected behavior of BR molecules in the human body or mammals in general, in which water is the main physiological medium. Simultaneously, because of the inactivity of the liver in the early days of the newborns, BR is expected to be present as small solid particles or as suspensions in the human body, especially if not bound to the HSA. This highlights the importance of studying BR in the solid form or as suspensions in proper solvents, such as water, to fully understand the reaction pathways of suspended BR particles before excretion under physiological conditions. Unfortunately, to date, there is no such photophysical study on the BR in the solid state.

Herein, we report, for the first time, a photophysical study on the BR in the solid state, by combining first principles calculations and time-resolved emission spectroscopy. We reveal unique strong emission properties of the BR crystals, and we rationalize these peculiar features on the base of the crystal structure. These unprecedented findings have been extended as well to suspensions in water. Understanding the behavior of BR in the solid state should provide information on the behavior of BR particles in the newborns before excretion, as well as on the overall picture of the excited-state dynamics of BR molecules inside human bodies under phototherapy procedure.

## Methods

### Chemicals

The solvents, acetonitrile and CHCl_3_ (both Sigma-Aldrich, spectrophotometric grade), MeOH (Sigma-Aldrich, Chromasolve), and distilled water were used without further purification. BR was purchased from Sigma-Aldrich as a mixed isomer sample and from Frontier Scientific Porphyrin Products.

### Steady-State Spectroscopy

Absorption spectra were measured on a Varian Cary 5000, and emission measurements were performed using a Horiba Jobin Yvon Fluorolog and automatically corrected for wavelength-dependent instrument sensitivity. The reflectance measurements have been measured for solid samples as well to accommodate for the expected scattering effect. For the sake of clarity, the absorption data are shown herein as there were no significant differences between transmission and reflectance data. Solution measurements were carried out at the right angle in a 1 cm cuvette, while glass films and tapes were measured using the front-face geometry (ca. 30°). In order to remove self-absorption effects, the peak optical densities were maintained below 0.1 at the excitation wavelength. The front-face position was used for samples on glass slides. Emission spectra were recorded at 400 nm excitation wavelength. To measure the emission from the solid particles, BR was dissolved in CHCl_3_ and then spread over a glass plate until the end of solvent evaporation (crystals β). In this procedure, small and fuzzy particles were highly expected to be formed. Also, BR solid particles were directly used from the used batch on a plastic tape by taking background measurements to detect the emission crystals α. Upon adding organic bases to the suspensions of BR crystals in solutions, small volumes of concentrated bases were utilized and added directly to the suspension solutions without prior dilution. Thus, the presented data upon adding organic bases show qualitative data than quantitative ones.

### Time-Resolved Measurements: Time-Correlated Singel Photon Counting (TCSPC) and Streak Camera

The streak camera setup has been described in detail previously.^21^ Briefly, the excitation of the sample with ultrafast laser pulses was performed using a frequency-doubled Ti:Sa oscillator (Coherent Mira) output (400 nm) at 76 MHz. A 1.0 cm cuvette was used (for suspensions), and the laser beam was directed into the cuvette close to the cuvette wall on the emission side, thus reducing the efficient cuvette length to 1–2 mm. Fluorescence at a right angle to the excitation was passed through a Bruker SPEC 250IS and onto the streak camera. The charge-coupled device (CCD) camera was used in the binning mode (2 × 2 pixels) to give a 512 × 512 pixel matrix. The global fit procedure has been used for the kinetic analysis, and all the obtained data are presented in Table 1. Also, amplitudes are calculated from the absolute integral area under the decay-associated spectra. Herein, the Instrumental Response Function (IRF) of the utilized pulse is about 5 ps.

**Table 1:** Extracted Emission Lifetimes of BR under Various Conditions Using the Global Fitting Procedure

environment	@550 nm/ps	@700 nm/ps
MeCN	4.3 ± 0.1	260 ± 4
MeOH	3.1 ± 0.2	250 ± 15
water	5 ± 1.1	265 ± 5
**α** crystals	25 ± 2.8	240 ± 10
**β** crystals	5 ± 1	140 ± 6

### Scanning Electron Microscopy and Powder X-Ray Diffraction Techniques

The morphology of the BR powders was characterized by field emission scanning electron microscopy (FE-SEM) (LEO 1550, Schottky FEG, ZrO/W cathode) in the secondary electron detection mode at an accelerating voltage of 300 V, an aperture size of 30 μm, zero tilt, and averaging 100 frames. The sample (powder) was spread thin on a regular conductive carbon tape-covered aluminum stub. The powders were imaged as is without sputtering any conductive layer on top. The sample (powder) was suspended in EtOH; the slurry was homogenized briefly with a mortar and pestle, spread on a silicon sample holder, and allowed to dry at ambient conditions (shielded from strong light sources). Crystal structure characterizations were performed by powder X-ray diffraction (PXRD), with a Bruker D8 diffractometer using CuKα1 radiation, λ = 1.5406 Å, in the Bragg-Brentano configuration equipped with a LYNXEYE linear detector. The measurements were made using a 2θ range of 2–40°, with a rotating sample holder.

### Computational Details

All theoretical calculations were conducted at the density functional theory (DFT) level within the wB97XD functional^22^ and 6-31G(d) as the basis set. The choice of this functional has been motivated because of its capability to properly describe charge-transfer (CT) states by means of long-ranged correlations and dispersion effects via Grimme D2 empirical correction. The geometries of the monomers and aggregates (pentamers) conforming the crystal structure were extracted from the available X-ray diffraction (XRD) data.^23^ For these systems, the calculations were performed in the gas phase, whereas for the BR molecules in solution, the solvent environment was accounted for by the conductive polarizable continuum medium (CPCM) method.^24^ The excited-state properties were estimated by means of time-dependent DFT (TD-DFT) formalism and following the same level of theory as the one used for the ground state. The assignment of the exciton localization along the two fragments conforming the BR molecule (see Figure S1) has been performed by employing the transition density matrix manipulation tools implemented in TheoDORE package.^25^ One the same foot, natural transition orbitals (NTOs) were used to obtain a spatial representation of the lowest energy excitations.^26^ All sets of calculations were performed with the Gaussian 16 package.^27^

### Estimation of the Förster Resonance Energy-Transfer (FRET) Properties

The dependency of the relative position of the neighboring monomers M2 and M1 (see Figure S5) has been accounted for by means of the orientation factor κ, which is calculated as follows
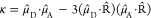
1where μ_D/A_ represents the normalized transition dipole moment of the donor/acceptor (M2/M1) monomers, and *R* is the normalized vector connecting the centers of mass for both monomers. Once we calculate the orientation factor κ, we can determine the Förster distance of this pair, the donor/acceptor pair, by following eq 2
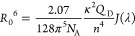
2
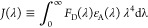
3where *N*_A_ is the Avogadro number, *Q*_D_ is the fluorescent quantum yield of the donor (M2), which is equal to 3 × 10^–4^ according to the experimental measurement, *n* is the reflective index of the medium set to 1.335, and *J*(λ) is the spectral overlap integral between the normalized donor M2 emission spectrum and the acceptor molar extinction coefficient (ε_A_)_max_ = 60,000 M^–1^ cm^–1^, as obtained from the M1 absorption spectrum. These parameters lead to a *J*(λ) value equal to 2.05 × 10^15^ M^–1^ cm^–1^ nm^4^.

## Results and Discussion

**Figure 1:**
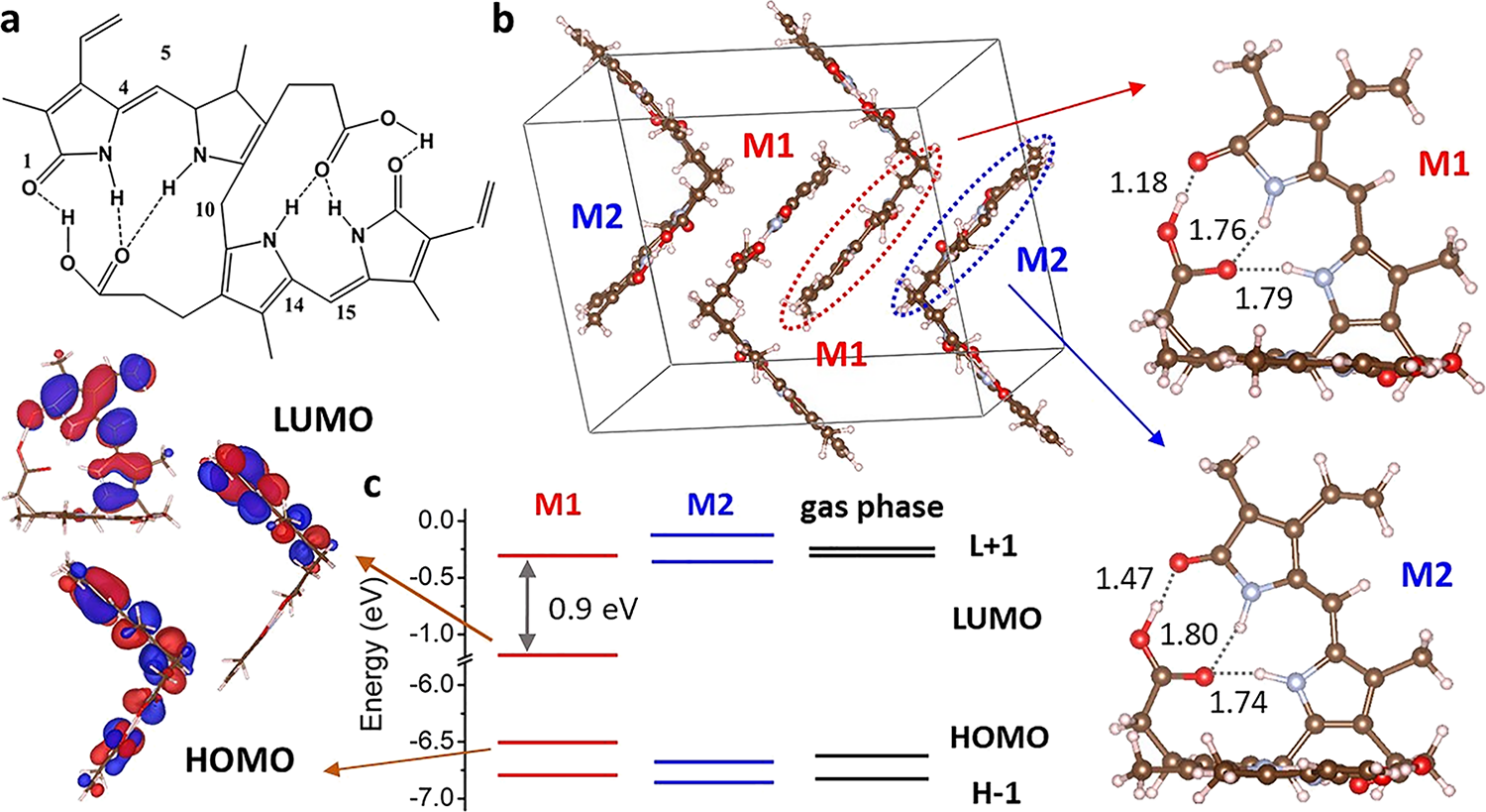
(a) Structure of the Z,Z BR isomer, showing the internal hydrogen bonds. (b) Perspective view of the unit cell forming the BR crystals made of two pairs of monomers (M1 and M2), which are displayed on the right part of the image. The H-bond distances highlighted here are given in Å. (c) Frontier energy levels for M1 (red), M2 (blue), and BR in the gas phase (black), with the isodensity plots of the LUMO and HOMO of M1 represented in the left part of the graph. The rest of the frontier molecular orbital isodensity plots are collected in Figure S3. The isovalue used to plot the isodensities was 0.02 a.u.

Figure 1a shows the chemical structure of BR, consisting of two almost equivalent units, that is, the *endo* and *exo* forms of a dipyrrinone chromophore, linked by a saturated CH_2_ group. The two halves also form strong internal hydrogen bonds (H-bonds) that can be broken upon illumination through a fast isomerization process, making the isomerized BR vulnerable to interact with water and special proteins to be excreted easily through the bile.^19^ The most stable isomer form of BR is called Z,Z-BR, which is insoluble in most solvents, including water, because of the strong internal hydrogen bonds.^28^

**Figure 2:**
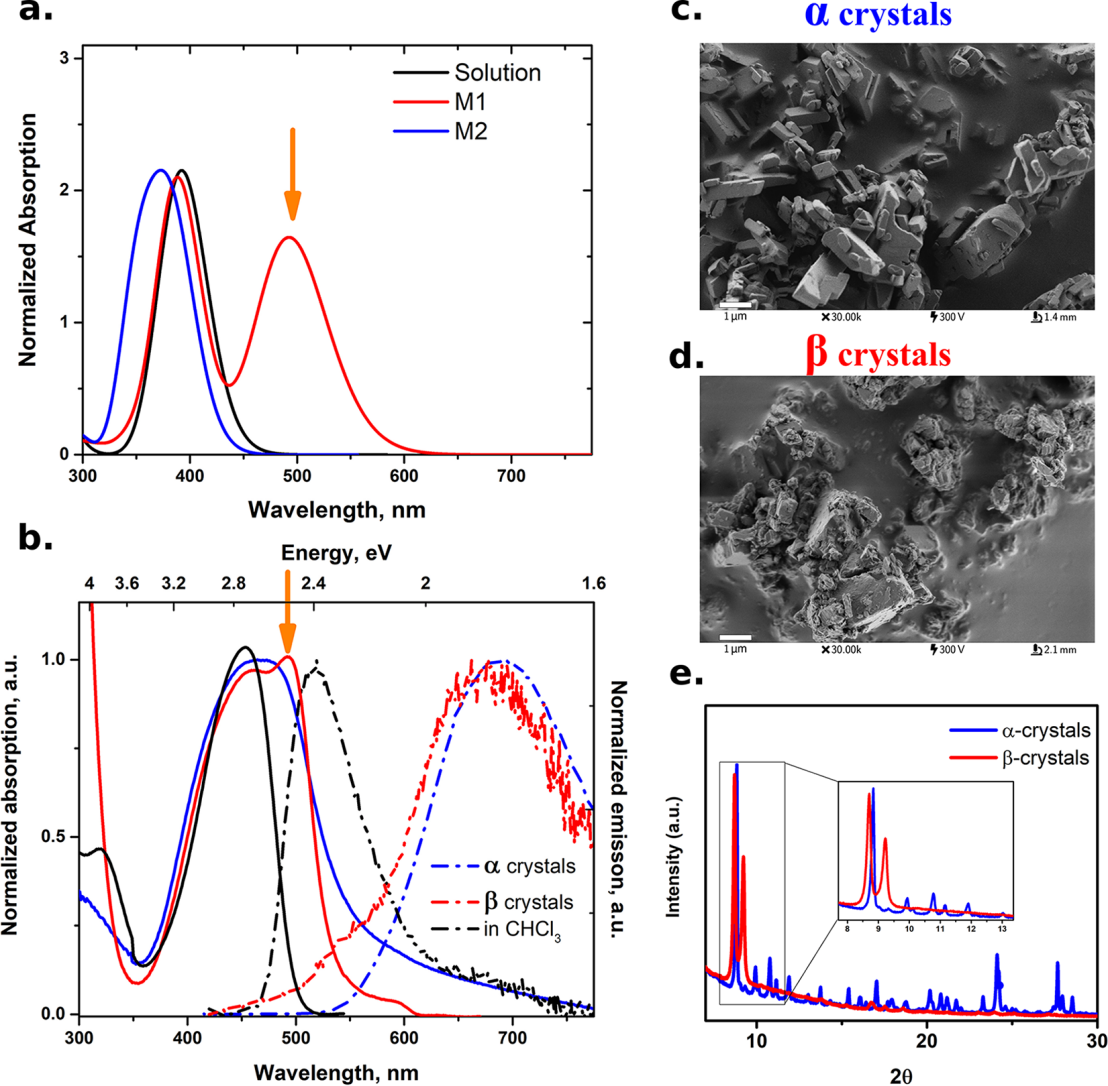
(a) Calculated absorption spectra of M1 and M2. The nature of the main transitions is reported in Table S2. (b) Experimental absorption and emission of BR in solution and solid state. SEM images for α crystals, M1, M2 (c), and β crystals mainly M1 (d). (e) XRD measurements for α and β crystals of BR. The XRD data show many isomers in the crystal package. Most of “M2” isomers have been disappeared after rough recrystallizations because of their solubility or their conversion into another isomer “check the new intense peak close to the potential M1 peak.”

The crystal structure package, based on the previous XRD results of BR crystals,^23^ shows that two different BR units are present, hereafter indicated as M1 and M2 (see Figures 1b and S1). Although the BR monomers may appear quite similar, one fragment of M1 shows an internal strong H-bond between the O atoms (1.18 Å), which is absent in the M2 fragments (see the right part of Figure 1b). This H-bond is very strong, close to be a real covalent bond, with a distance of 1.18 Å (see also Figure S1 for other fragments). This strong H-bond is also expected to make M1 monomers more restricted and less flexible toward any excited-state large-scale motion and more insoluble in various solvents with respect to M2. Interestingly, DFT calculations performed on the X-ray structures of M1 and M2 disclose that the two highest occupied and lowest unoccupied molecular orbitals (HOMO – 1/HOMO and LUMO/LUMO + 1) are not-longer quasi-degenerate for M1 (see Figure 1c). Indeed, the structural distortion engenders a sizable stabilization (∼ 0.9 eV) of the LUMO level, being localized on the distorted moiety (see left part Figure 1c**)**, and a slight destabilization of the HOMO level. Consequently, the calculated absorption spectrum (see Figure 2a and Table S2) presents two distinct absorption peaks, centered around 390 and 490 nm, corresponding to local fragment excitation from the HOMOs to LUMO + 1 (S_2_) and to LUMO (S_1_), respectively (see Figure S3–4). On the other hand, a single absorption band is calculated for M2, around 385 nm, because of the near-energy degeneracy of the LUMO and LUMO + 1 (Figure 1c). Thus, the absorption spectrum of the crystal package of BR is expected to be a combination of M1 and M2 spectral bands. Note that the absorption features of the monomers are not substantially modified upon interaction with the neighboring molecules of the crystal (see Table S3). We called this crystal package “α crystals.” Interestingly, the simulated spectrum of BR in CHCl_3_, after full structural relaxation, exhibits a single absorption peak around 395 nm, which is very close to the calculated maximum absorption of M2 (see Figures 1c and 2a), as expected on the basis of their similar geometrical and electronic properties (see Section 1.1 in the Supporting Information). This indicates that M1 can only be identified in the solid-state BR, where these strong H-bonds can be formed thanks to the crystal packing.

To verify these theoretical observations, absorption and emission of BR α crystals have been studied in crystalline solid form and after dissolution in CHCl_3_ (see Figure 2b). Upon dissolving the BR crystals in CHCl_3_, it has been noticed that the solution is not fully clear and a simple filtration step is needed to obtain a clear solution because of the insolubility of M1. The filtered solution in CHCl_3_ shows a strong absorption band centered at ca. 450 nm, with a corresponding weak emission band at 520 nm, matching with the previous measurements of BR in CHCl_3._^10−12,14^ However, the BR α crystals (see the SEM image for large crystals, Figure 2c) show a broader absorption band centered at ca. 475 nm, with a significant absorption tail extending to 750 nm (blue solid and dashed lines in Figure 2b). Interestingly, the absorption band of α crystals matches quite well with that of soluble BR in CHCl_3_ (full and dashed black lines in Figure 2b) in the high-energy side of the spectrum. Surprisingly, the emission of α crystals shows a stronger red-shifted band centered at ca. 700 nm. To the best of our knowledge, this observation has not been detected previously for the BR molecules. However, based on our quantum chemical calculations, we can interpret these spectral differences between BR in CHCl_3_ and BR in the solid state as arising from the contribution of M1 monomers. Indeed, while in the BR α crystals, both M1 and M2 forms are present, and the filtered solution may not contain M1 because of its lower solubility caused by the strong intramolecular H-bond mentioned above. To indirectly verify this assumption, we dissolved BR α crystals in CHCl_3_, removed most of the clear solution, and re-evaporated CHCl_3_, forming the BR crystals again. In this way, we expect to have less M2 monomers in the formed crystals because most of them have been removed by dissolution in CHCl_3_. The BR crystals have been imaged by SEM before and after this rough recrystallization process (see Figure 2c,d). Clearly, as shown in the SEM images, this recrystallization process is not optimum, but still the crystallinity of the BR particles is present, as shown by the XRD measurements (see Figure 2e). These new BR crystals are named “β crystals.” As expected, the absorption and the emission properties of the two BR crystal forms are different (see the orange arrow in Figure 2b). The absorption band of the β crystals resembles that of the α crystals, but a clear absorption peak at 500 nm is present. This low-energy absorption peak matches with the calculated lower-energy absorption band for the M1, which is localized on the “distorted” dipyrrinone unit (see Figure 2a,b). On the other hand, the measured emission of the β crystals is slightly different. Although the main emission peak at ca. 700 nm is still present, the emission is weaker, and there is the appearance of a new emission shoulder at roughly 525 nm. Interestingly, the position of this new emission shoulder almost coincides with the emission band of soluble BR in CHCl_3_ (see Figure 2b).

**Figure 3:**
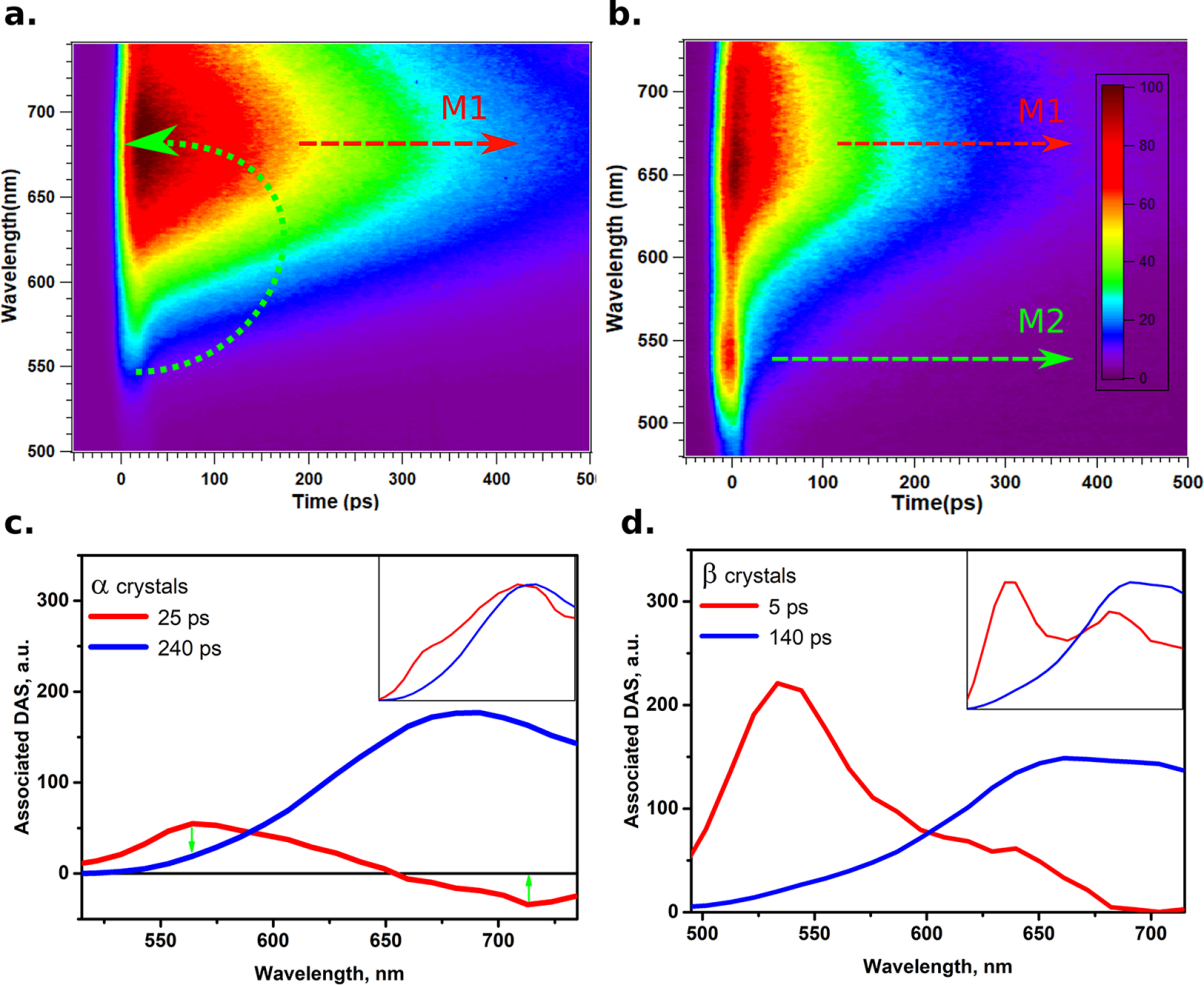
(a) Time-resolved emission properties of BR α crystals. (b) Time-resolved emission of BR β crystals. (c) Decay-associated spectra for α crystals for two emission lifetimes. In the inset, the extracted normalized emission spectra at corresponding times 25 ps (red) and 240 ps (blue) are shown. (d) Decay-associated spectra for β crystals for two emission lifetimes. In the inset, the extracted normalized emission spectra at corresponding times 5 ps (red) and 140 ps (blue) are shown. See text for more information.

To fully rationalize these peculiar spectral properties, especially the emission ones, we utilized time-resolved emission with picosecond time resolution. The BR α crystals show long-lived emission at room temperature with a corresponding emission lifetime of 240 ps (see Figure 3a). Knowing that the EQY of BR in the solution is about 0.03% (average emission lifetime is about 0.3 ps),^12,20^ the EQY for the BR α crystals is about three orders of magnitude higher (∼24%). This high EQY of BR α crystals is possibly due to restriction of the ultrafast isomerization process of BR, which increase the barrier for the conical intersection in the excited state, as previously shown in similar systems.^10−12,29,30^ Moreover, we interpret this red-emission peak as coming from M1, whose S_1_ state is lower in energy with respect to M2 (492 vs 384 nm for M1 and M2, respectively). Then, we report transient-emission data measured on the BR β crystals in Figure 3b. First, the emission peak at ca. 700 nm has a shorter lifetime of 140 ps, possibly due to the surface defects formed during the recrystallization process.^31−33^ Second, a clear short lifetime emission shoulder at 525 nm appears, with an average lifetime of 5 ps (EQY ∼0.5%) (see Table 1**)**. We assign this short emission peak, which coincides with the emission of BR in solution, to the M2 monomer emission present in the β crystals. Interestingly, the EQY of M2 in β crystals is similar to that of BR upon binding to HSA, indicating that the isomerization of M2 in β crystals is partially restricted.^12,20^

The question that we need to answer now is why the green emission shoulder does not appear in the well-packed α crystals. To validate our assignments and explain the differences in the emission dynamics, we have explored the possibility of realizing Förster resonance energy transfer (FRET) from M2 to M1 among the very close and well-packed different BR molecules in the α crystals (see details in section 1.6 in the Supporting Information). Herein, invoking a FRET mechanism is not exotic because, previously, FRET was also suggested to occur between the two halves of BR (XBRs) in solutions and then disappear in the XBR molecule, as well as upon deprotonation of BR.^10^ Indeed, FRET rates are dependent on the relative position of the donor–acceptor pairs, which is expressed as follows^34^
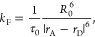
4where τ_0_ is the lifetime of the donor excited state, *R*_0_ is the Förster radius, *r*_D_ and *r*_A_ are the position vectors of the donor (M2) and the acceptor (M1), respectively. The Förster radius, defined as the distance between the donor and the acceptor when the FRET has 50% probability, can be calculated from the donor luminescence efficiency, the overlap integral of the donor emission spectrum and the acceptor absorption spectrum, and the dimensionless orientation factor κ,^2^ which can vary from 0 to 4 (see the Methods section for more details). The calculated κ^2^ values for the four possible combinations of neighboring M2–M1 pairs present in the crystalline structure (see Figure S5 and Table S4) amount to 0.07, 0.86, 0.78, and 2.2. This result manifests an anisotropic transport, with one of the donor–acceptor pair energy transfer being clearly unfavorable with respect to the others. Notably, these values of κ^2^ yield to donor/acceptor pair Förster radii values of 10.2, 15.6, 15.4, and 18.3 Å, being of the same order of magnitude of the FRET radii estimated in common energy-transfer processes occurring in biological systems (i.e., *R*_0_ values for naphthalene–dansy pairs amounted to 23.3 Å).^35^ Thus, these results corroborate the hypothesis that long-range energy-transfer processes may take place among neighboring M2 and M1 monomers in the α crystal arrangement. The FRET process is also confirmed through the decay-associated spectra (DAS) obtained by fitting the time-resolved data, as shown in Figure 3c,d.

For the α crystals, two emission lifetimes are needed for the global fitting, a short lifetime of 25 ps and a longer lifetime of 240 ps. The DAS for the latter shows an emission-like behavior, as expected. On the other hand, the DAS for the short-lifetime component shows a positive signal around 570 nm and a negative part centered at 715 nm. This negative part is an indication for the FRET process between the emission components at the blue side (M2) to the ones at the red side (M1) of α crystals through the absorption of M1 monomers.^36−38^ The ratio between the positive and the negative parts of DAS for 25 ps component indicates that the FRET efficiency is at least 60–70%. For the β crystals, the DAS of the short component (∼5 ps) shows only an intense positive part extending to 675 nm, indicating the absence of the FRET process between the blue and red-emission sides. Thus, we expect that, upon recrystallization, the ordered crystalline packing is broken, hindering efficient FRET processes between M2 and M1 and thus facilitating the appearance of M2 emission at ∼525 nm.

**Figure 4:**
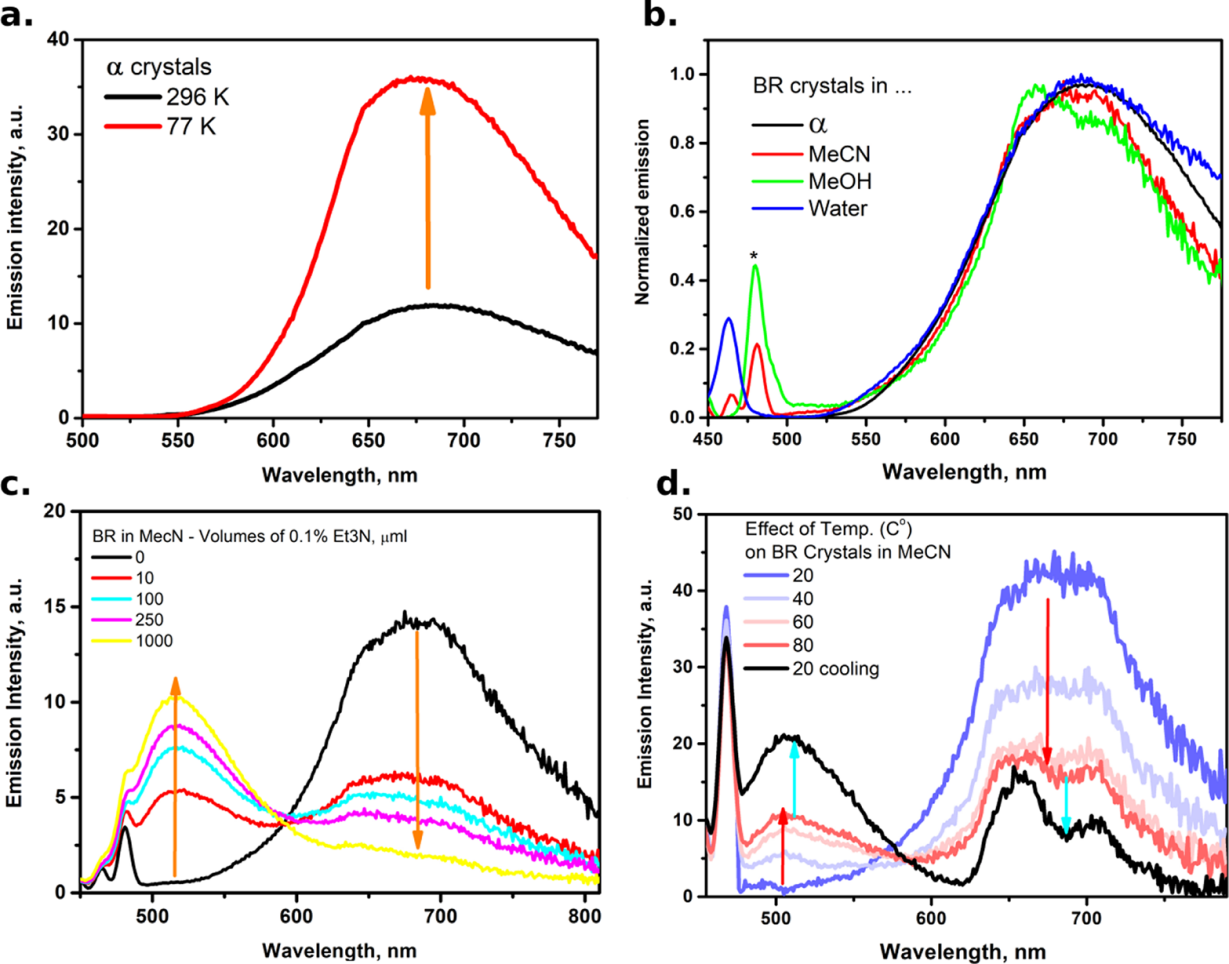
(a) Effect of decreasing the temperature on the emission properties of α crystals. (b) Emission spectra of suspended BR crystals in various solvents, as shown in the legend. The star shows the Raman lines of solvents. (c) Addition of organic base Et_3_N to the suspension of BR crystals in MeCN. (d) Effect of heating and cooling the suspensions of BR in MeCN. See text for more information.

To determine the amount of active nonradiative channels, the emission of BR crystals has been measured at low temperature (77 K), and, as it is apparent in Figure 4a, the emission intensity increases by a factor of 3, meaning that the EQY can reach ∼75%. This indicates that nonradiative decay channels are still active at room temperature for the α crystal, suggesting a residual activity of large-scale motions,^20,29,30,39−42^ aggregation effects,^21,29,43^ or heat dissipation by crystal phonons.^44,45^

To correlate the behavior of BR in the crystal form and the one in the physiological environment, we performed emission measurements for suspensions of BR crystals in water and other polar solvents, such as MeOH and MeCN (see Figure 4b). All the emission data show results that are consistent with those measured in the solid phase, indicating that the photophysical properties of BR inside the human body can be rather safely interpreted on the basis of these results. Interestingly, there is also a conversion process between the insoluble M1 and soluble M2 monomers upon changing the basicity of the environment or inducing a thermal effect. Adding small volumes of an organic base (trimethylamine, Et_3_N) to the suspensions of BR in MeCN shows a systematic suppression of the red-shifted emission and an evolution toward the well-known green emission of BR in the solution. Adding the organic base is expected to deprotonate the BR monomers, breaking the internal strong H-bonds.^29,40,46^ Such an effect of equilibrium between M2 and M1 monomers depends on the solvent as well as on the strength of the employed base, as shown in Figure S6 in the Supporting Information. This is the consequence of the rupture of the strong H-bonds in M1 and the formation of soluble M2 monomers in the solution. Similarly, by increasing the temperature of the suspended BR crystals in MeCN, the red emission from M1 monomers decreases, while the green emission of M2 monomers increases. Moreover, this thermal effect is not reversible; thus, cooling the suspensions again to room temperature does not regain the red emission because of the continuous breakdown of the H-bonds and the loss of the symmetry of the crystal packing (see Figure 4d).

**Scheme 1:**
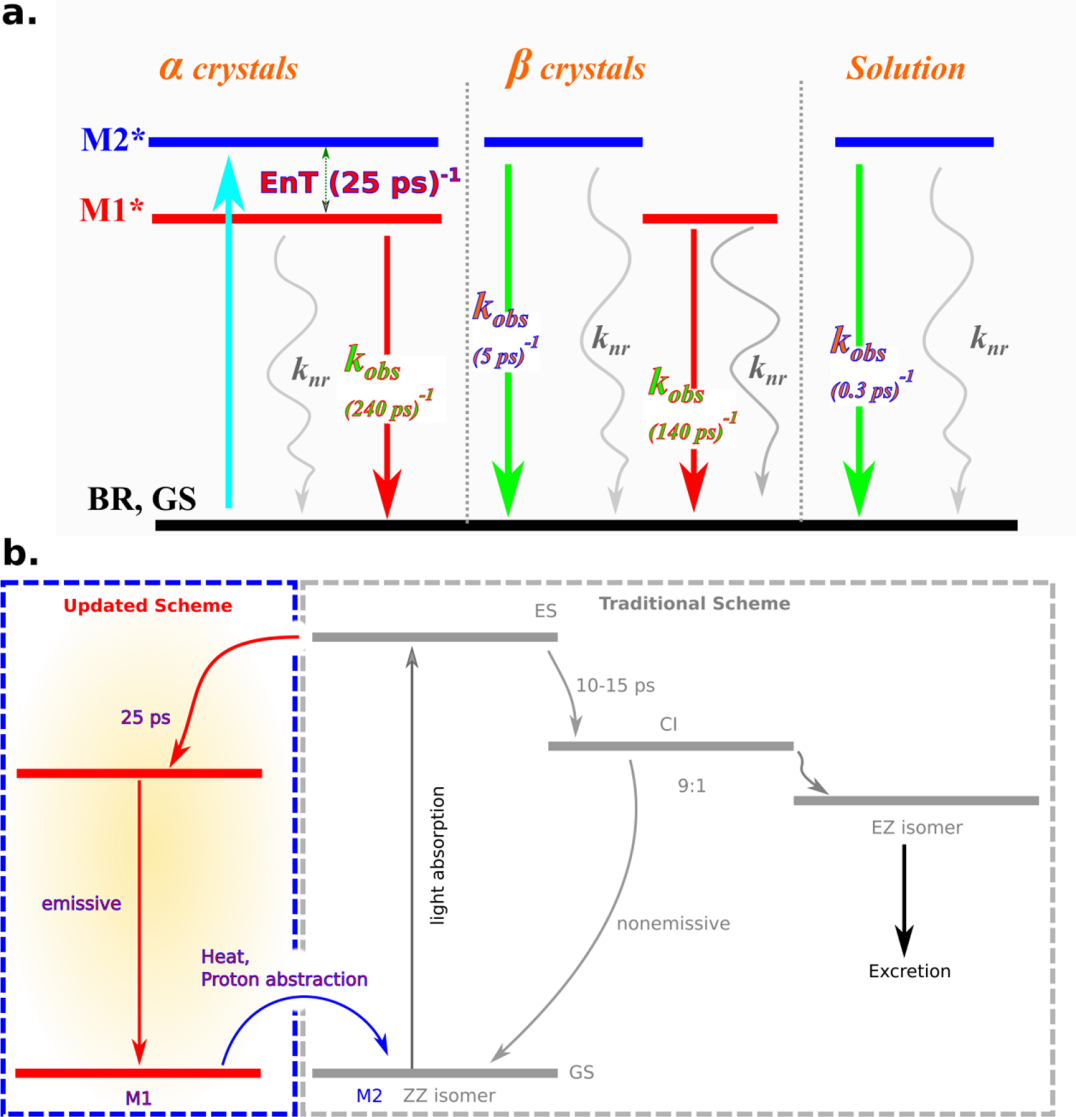
(a) Schematic Representation of the Possible Photophysical Processes in the Solid-State Crystals of BR Crystal versus That in the Solution and (b) Schematic Representation of the Old and the Updated View for the Photoexcited State of BR upon Interaction with the Incident Light in the Human Body

Scheme 1a summarizes the current photophysical findings for BR under three different phases. In the well-packed crystals, α crystals, BR is present in the form of M1 and M2 monomers, having different geometries, and electronic properties. Upon light absorption, an efficient FRET process, with a lifetime of ∼25 ps, occurs from M2 to M1, making M1 responsible for the main red-shifted emission at 700 nm with a QY close to 24% (τ_obs_ 240 ps). For the loosely packed β crystals, the energy transfer is hindered and a dual emission, at ∼525 nm (τ_obs_ ∼ 5 ps) and at ∼700 nm (τ_obs_ ∼ 140 ps), is measured. When BR is completely soluble, only emission at 525 nm can be observed, with an EQY of 0.03% (τ_obs_ 0.3 ps) because of the well-known isomerization process. Apparently, the isomerization process and, possibly, other nonradiative deactivation processes are still efficient in the solid state because the BR crystal EQY cannot reach unity even at low temperatures (see Figure 4a). Moreover, in suspensions, by changing the temperature or controlling the environment basicity, one can break the strong intramolecular H-bond characterizing M1, thus obtaining symmetrical M2 monomers and controlling the emission properties.

Despite the effectiveness of the phototherapy to treat excess levels of BR,^8,47^ these present findings may shed new light on the current understanding of the phototherapy for the excretion of BR. Previously, no significant efficiency differences have been detected upon using blue light (∼460 nm) or green light (∼500 nm).^48^ As mentioned before, all the previous studies on BR focused on the BR in solution, which is on the “M2” monomer in our scheme, and this leads to the picture about the current mechanism, reported in Scheme 1b, for the interaction of M2 with incident light. In this picture, M2 reaches the conical intersection (CI) within 10–15 ps, and then, isomers of a different ratio 9:1 (ZZ:EZ) are formed.^10−12^ Inserting M1 monomers into the same picture opens another deactivation channel (FRET to M1) for the excited M2 monomers, thus decreasing the efficiency to form excretable EZ isomers. Because M1 monomers have strong internal H-bonds and are highly hydrophobic, absorption of light by M1 monomers is expected to be useless rather than the expected red emission, which might provide the jaundice color for infants. As we have shown, M1 can be converted into excretable M2 monomers by heating because of the incident light or proton abstraction by some proteins. However, these are expected to be slow processes, ∼hours to days, thus requiring prolonged exposure to irradiation, with the correlated higher risks for newborns’ health, such as risk of skin cancer, as it is the case in the current phototherapy protocol.^8,49^ However, further investigations in vivo are needed to confirm this hypothesis.

## Conclusions

Despite the biological relevance of BR, and the understandable great interest devoted to characterize its photophysics in the past years, previous studies have only addressed its absorption and emission properties in solution, which, however, does not mimic the actual biological environment, where BR is likely to be present as small solid particles or as suspensions when not bound to the HAS. Here, for the first time, we have shown that BR single crystals present a unique and completely different photophysical behavior with respect to that reported in the literature. The analysis of the BR crystal (α crystals) structure and quantum chemical calculations discloses those in the solid-state phase; BR is present in two different monomeric units, namely, M1 and M2. Particularly, strong internal hydrogen bonds make M1 very poorly soluble and slightly distorted in the crystal packing with respect to M2 and to the BR structure in the solution. This structural distortion leads to a sizable stabilization of the lowest unoccupied energy levels in M1 and, thus, to a marked shift at longer wavelengths of both absorption and emission, occurring at ca. 700 nm with a high EQY of ca. 24%. When dissolving BR α crystals, the more soluble M2 molecules are dissolved, and after the irradiation of the filtered solution, the well-known and efficient isomerization process decreases the EQY to 0.03%. We also obtained different BR crystals (β crystals), mainly composed of the less-soluble M1 units, after the dissolution of α crystals in CHCl_3_, elimination of most of the clear solution, and solvent re-evaporation. The different crystal forms have strikingly different photophysics, that we have shown, based on theoretical calculations, originate from the possibility of realizing efficient long-range energy-transfer (FRET) processes from M2 to M1 in the original α crystals, solely leading to the emission at 700 nm. Inefficient FRET in β crystals gives rise to dual emission, at ca. 525 and 700 nm, from M2 and M1, respectively. Similar photophysical properties have been detected for BR particles in water as a physiological environment, suggesting that the observed photophysical properties in the solid state can also occur under the phototherapy procedure. Thus, we suggest a more complex phototherapy scheme of BR, which may explain the prolonged exposition to light irradiation that is required for an effective treatment. Therefore, our study opens a new dimension for understanding the BR excited-state dynamics for an efficient phototherapy.

## Supporting Information Available

Lateral views of the two fragments A and B conforming the BR molecules; lateral and perspective views of the superimposed M1 (red) vs M2 (blue), M1 vs ground state (GS), and M2 vs GS (black) structures; root-mean-square-displacement values of the internal coordinates; frontier molecular orbital shapes for M1, M2, and BR optimized in the gas phase; excitonic properties for the lowest-energy transitions bands of the simulated spectra; NTOs for the two lowest energy excitations of M1, M2, and BR molecules in the CHCl_3_ solution; top views of the four different nearest neighbor pentamers; excitonic properties for the lowest-energy transitions of the monomer (gray) and pentamer (yellow) conformations; and orientation factors (κ^2^) and Förster distances (*R*_0_) for the donor/acceptor pairs (PDF)
