# Supplementary material for: Highly Emissive Biological Bilirubin Molecules: Shedding New Light on the Phototherapy Scheme
Source: J Phys Chem B. 2021 Aug 4;125(32):9213–22. doi: 10.1021/acs.jpcb.1c05308 (PMC8389986; doi:10.1021/acs.jpcb.1c05308)
Supplement: Supplementary file 1 — jp1c05308_si_001.pdf [file jp1c05308_si_001.pdf]

Supporting Information

Highly Emissive Biological Bilirubin Molecules: Shedding new light on the Phototherapy Scheme

Ahmed M. El-Zohry,[a,b,*] Valentin Diez-Cabanes,[c] Mariachiara Pastore,[*c] Taha Ahmed,[a] and Burkhard Zietz [a]

[a] Department of Chemistry - Ångström Laboratories, Box 523, SE-75120 Uppsala, Sweden,
[b] Department of Physics - AlbaNova Universitetscentrum, Stockholm University, SE-10691 Stockholm, Sweden.
[c] Université de Lorraine & CNRS, Laboratoire de Physique et Chimie Théoriques (LPCT), F-54000, Nancy, France
* ahmed.elzohry@fysik.su.se, amfzohry@yahoo.com, mariachiara.pastore@univ-lorraine.fr

1. Supporting Figures and Tables

1.1. Geometrical properties BR molecules

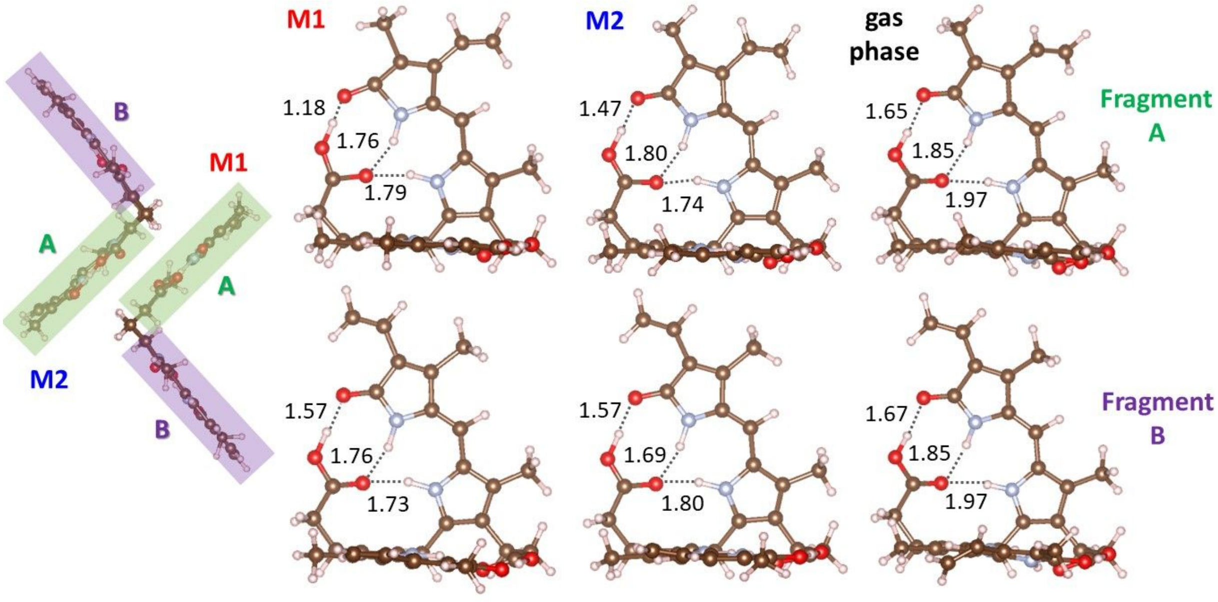

Figure S1: a) Lateral views of the two fragments A and B conforming the BR molecules (M1 and M2 monomers and BR relaxed in gas phase), as schematized in the right part of the graph. Dashed lines are used to represent the H-bonds, whose distances are given in Å units.

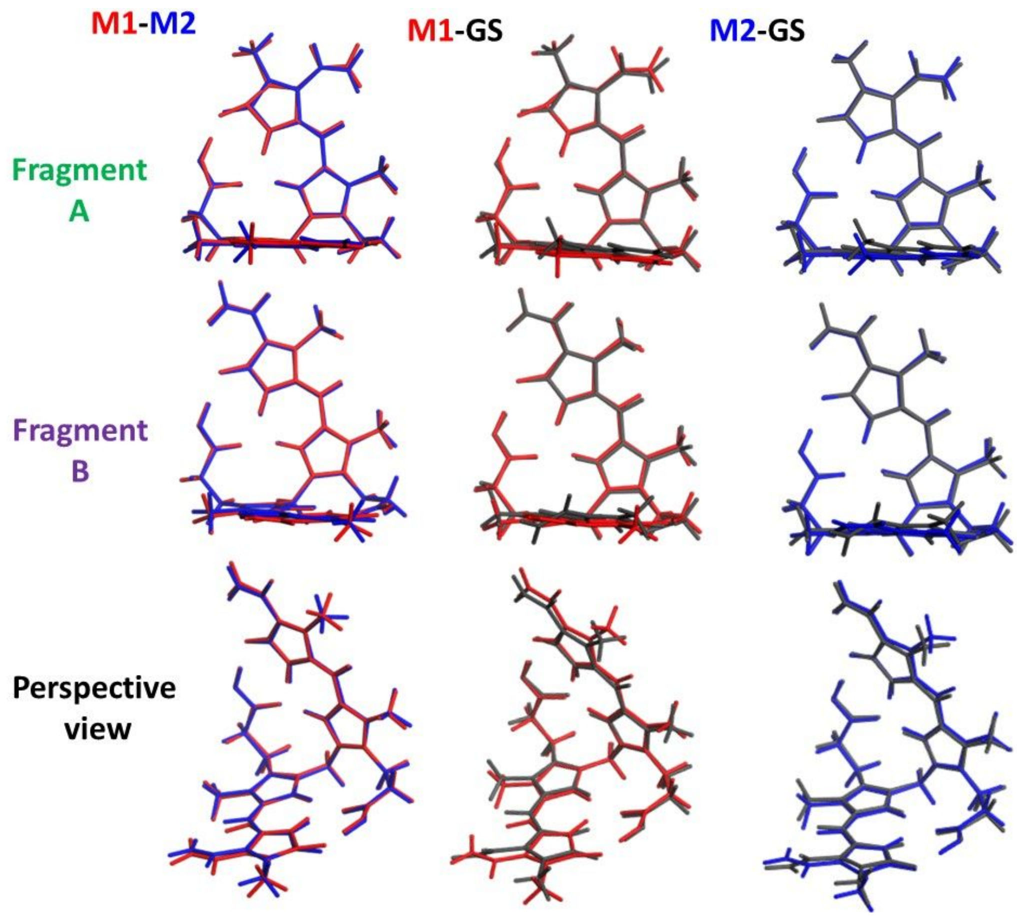

Figure S2: Lateral and perspectives views of the superimposed M1 (red) vs M2 (blue), M1 vs ground state (GS), M2 vs GS (black) structures (from the left to the right).

Table S1: Root-Mean-Square-Displacement (RMSD) values of the internal coordinates for pairs of BR molecules depicted in Figure S2.

Pairs	bonds (Å)	angles (^{0})	dihedral (^{0})
M1-M2	0.050	7.41	13.46
M1-GS	0.058	8.20	17.62
M2-GS	0.039	3.64	10.08

1.2. Electronic properties BR molecules

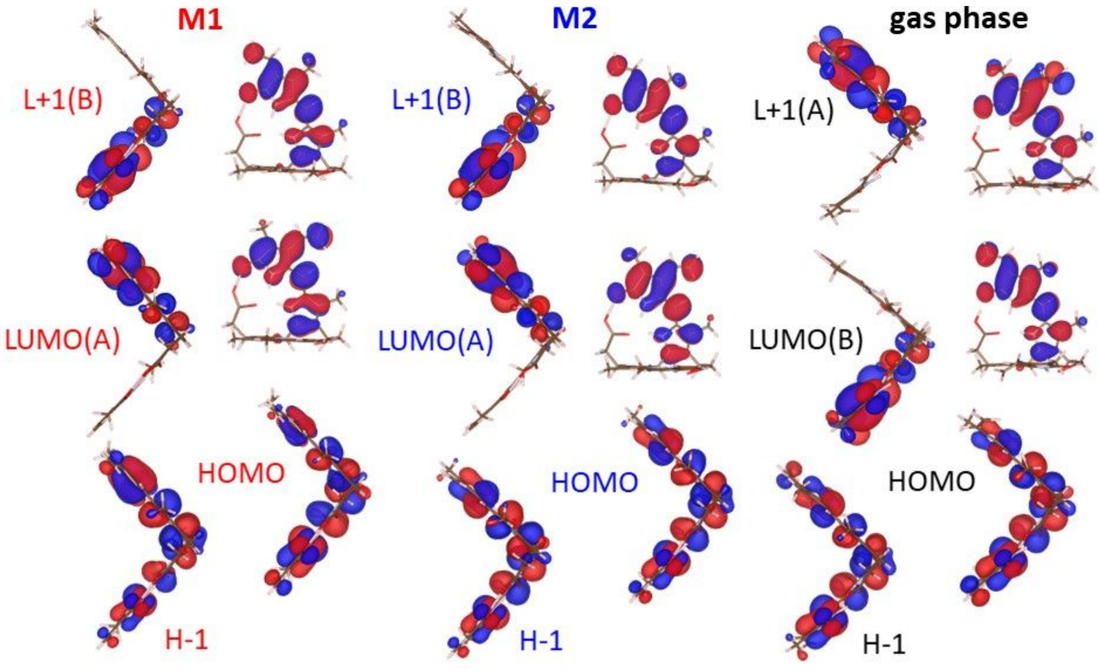

Figure S3: Frontier molecular orbital shapes for M1, M2 and BR optimized in gas phase (from the left to the right part). In the case of the unoccupied orbitals, the BR fragment where is localized the isodensity is indicated within parenthesis. The corresponding energy values of these orbitals are plotted in Figure 1-c. The isovalue used to plot the isodensities was 0.02 a.u.

1.3. Optical properties BR molecules

Table S2: Excitonic properties for the lowest-energy transitions bands of the simulated spectra represented in Figure 2-a: number of state (n), energies in eV, wavelengths (nm), oscillator strengths (*f*); and exciton localization along the fragments depicted in Figure S1.

	M1				M2				$CHCl_3$			
n	E_x (eV)	λ (nm)	*f*(a.u.)	*trans*	E_x (eV)	λ (nm)	*f*(a.u.)	*trans*	E_x (eV)	λ (nm)	*f*(a.u.)	*trans*
1	2.52	492	0.58	A→A	3.23	384	0.72	A→A	3.12	397	1.32	B→B
2	3.19	389	0.73	B→B	3.52	352	0.60	B→B	3.27	379	0.54	A→A
3	3.33	372	0.02	B→A	4.22	294	0.07	A→A	4.27	290	0.07	B→B
4	3.56	348	0.05	A→A	4.36	284	0.00	B→A	4.34	286	0.00	A→B
5	3.87	320	0.00	A→A	4.57	271	0.01	A→B	4.38	283	0.12	A→A

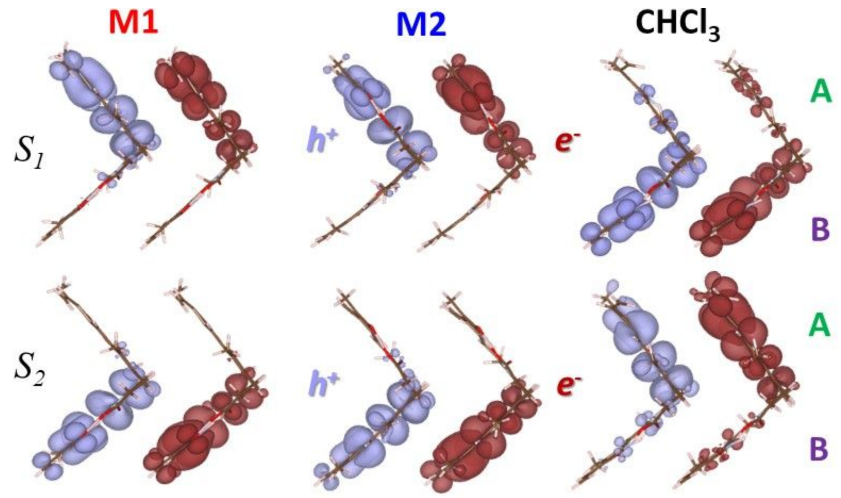

Figure S4: NTOs for the two lowest energy excitations of M1, M2 and BR molecule in $CHCl_3$ solution. Purple and red colors are used to represent hole and electron isodensities, respectively. The isovalue used to plot the isodensities was 0.02 a.u.

1.4. Optical properties BR pentamers

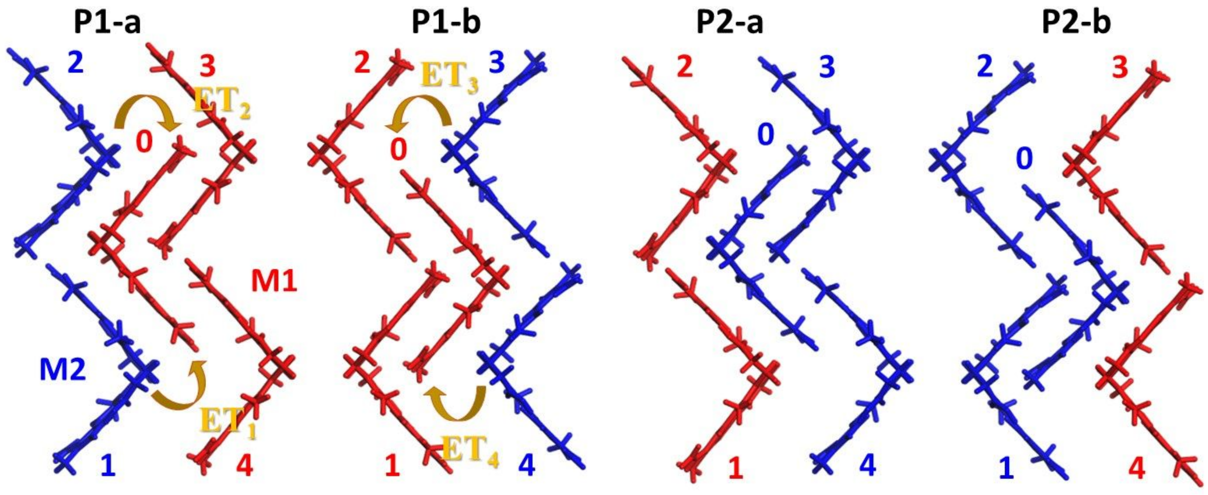

Figure S5: Top views of the four different nearest neighbor pentamers present in the crystalline phase, where P1 and P2 are used to name those structures where M1 and M2 are at the monomer located at the center. Yellow arrows represent the four possible combination of FRET processes between M2 donors and M1 acceptors.

Table S3: Excitonic properties for the lowest-energy transitions of the monomer (grey) and pentamer (yellow) conformations represented in Figure S5: number of state (n), energies in eV, wavelengths (nm), oscillator strengths (*f*); and exciton localization along the A_i and B_i fragments of each monomer *i*.

	M1-a				P1-a			
n	E_x (eV)	λ (nm)	*f* (a.u.)	*trans*	E_x (eV)	λ (nm)	*f* (a.u.)	*trans*
1	2.52	492	0.58	A→A	2.39	518	0.03	$A_0A_3 \rightarrow A_0A_3$
2	3.19	389	0.73	B→B	2.53	490	0.96	$A_3 \rightarrow A_3$
3	3.33	372	0.02	B→A	2.54	488	0.34	$A_4 \rightarrow A_4$
4	3.56	348	0.05	A→A	2.69	461	0.17	$A_0 \rightarrow A_3$
5	3.87	320	0.00	A→A	2.81	441	0.18	$A_3 \rightarrow A_0$
6	3.97	312	0.03	A→A	3.03	409	0.83	$A_2 \rightarrow A_2$
7	4.06	306	0.00	A→A	3.07	404	0.13	$B_0 \rightarrow B_0$
8	4.28	290	0.02	B→B	3.14	395	0.54	$A_1 \rightarrow A_1$
9	4.32	287	0.00	A→B	3.16	392	0.94	$B_4 \rightarrow B_4$
10	4.39	282	0.00	A→A	3.19	388	0.49	$B_3 \rightarrow B_3$
	M2-a				P2-a			
n	E_x (eV)	λ (nm)	*f* (a.u.)	*trans*	E_x (eV)	λ (nm)	*f* (a.u.)	*trans*
1	3.23	384	0.72	A→A	2.43	509	0.57	$A_2 \rightarrow A_2$
2	3.52	352	0.60	B→B	2.52	492	0.64	$A_1 \rightarrow A_1$
3	4.22	294	0.07	A→A	2.94	422	0.03	$A_0A_3 \rightarrow A_0A_3$
4	4.36	284	0.00	B→A	3.07	403	0.28	$B_1 \rightarrow B_1$
5	4.57	271	0.01	A→B	3.1	401	0.00	$A_0 \rightarrow A_2$
6	4.62	268	0.01	B→B	3.12	398	1.26	$A_3 \rightarrow A_0A_3$
7	4.85	256	0.06	B→B	3.14	394	0.56	$B_1 \rightarrow B_1$
8	4.89	254	0.00	B→B	3.18	390	0.46	$A_4 \rightarrow A_4$
9	4.91	252	0.09	A→A	3.18	390	0.26	$B_2 \rightarrow B_2A_2$
10	4.96	250	0.00	A→A	3.37	368	0.00	$B_1 \rightarrow A_1$

Table S4: Orientation factors (κ^2) and Förster distances (R_0) for the donor/acceptor pairs represented in Figure S5.

ET_i	κ^2	R_0 (Å)
1	0.065	10.2
2	0.855	15.6
3	0.781	15.4
4	2.202	18.3

1.5. Solvent effects in BR emission

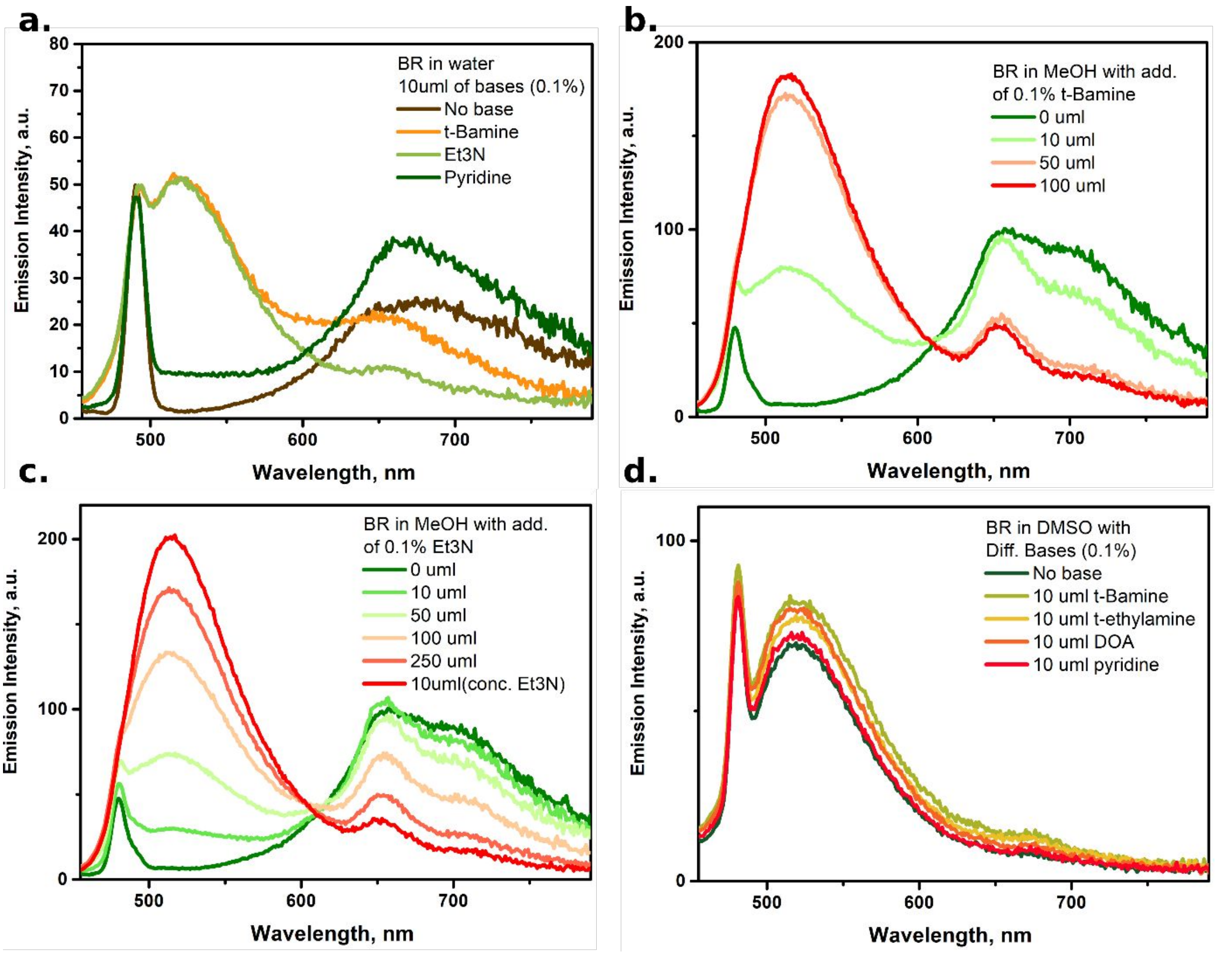

Figure S6: Suspension of BR in various solvents with addition different organic bases
